# Features of the Asynchronous Correlation between the China Coal Price Index and Coal Mining Accidental Deaths

**DOI:** 10.1371/journal.pone.0167198

**Published:** 2016-11-30

**Authors:** Yuecheng Huang, Wuyi Cheng, Sida Luo, Yun Luo, Chengchen Ma, Tailin He

**Affiliations:** 1 Center of Safety Research, China University of Geosciences, Beijing, China; 2 School of Engineering and Technology, China University of Geosciences, Beijing China; 3 School of Environment, Education and Development, University of Manchester, Manchester, United Kingdom; 4 School of Mechanical Engineering and Automation, Beihang University, Beijing China; Tianjin University, CHINA

## Abstract

The features of the asynchronous correlation between accident indices and the factors that influence accidents can provide an effective reference for warnings of coal mining accidents. However, what are the features of this correlation? To answer this question, data from the China coal price index and the number of deaths from coal mining accidents were selected as the sample data. The fluctuation modes of the asynchronous correlation between the two data sets were defined according to the asynchronous correlation coefficients, symbolization, and sliding windows. We then built several directed and weighted network models, within which the fluctuation modes and the transformations between modes were represented by nodes and edges. Then, the features of the asynchronous correlation between these two variables could be studied from a perspective of network topology. We found that the correlation between the price index and the accidental deaths was asynchronous and fluctuating. Certain aspects, such as the key fluctuation modes, the subgroups characteristics, the transmission medium, the periodicity and transmission path length in the network, were analyzed by using complex network theory, analytical methods and spectral analysis method. These results provide a scientific reference for generating warnings for coal mining accidents based on economic indices.

## Introduction

There is a significant correlation between work safety accidents and economic factors [1], and in China’s coal mining industry, economic factors are the primary cause of accidents [2–4] and can be divided into two aspects: safety investments made by associated enterprises and the macroeconomic performance of the coal mining industry. Although safety investments are mandated by relevant laws and regulations imposed by the government, such investments are not effective and ignored by most Chinese coal mining enterprises when they are in conflict with economic profits (as impacted by the coal economy). The macroeconomic performance factor can be divided into a number of specific factors, although most of these factors have not been thoroughly investigated. However, the correlation rules, especially the asynchronous correlation rules, between accidents and certain specific factors (e.g., coal prices) could provide a reference for a coal mining accident pre-warning system [5,6]. Asynchronous correlations are defined as a close relationship between two variables in asynchronous scenarios, and the time lag between these variables could be regarded as the period of time for pre-warnings. Thus, we can utilize economic factors to provide earlier and more accurate predictions of coal mine accidents. Therefore, we have studied the potential rules of the asynchronous correlation between coal prices and coal mine accidents.

In previous studies, a relationship has been observed between coal mine accident deaths and coal industry economic factors. Statistical theories and methods have been used to confirm that a Granger causality [6] cointegration relationship [5] occurs between coal mine deaths and coal prices. Coal prices have been shown to be a cause of coal mine accident deaths based on the asynchronous correlation between the two variables. However, the sample data selected in the research studies described above are monthly and/or annual time series data over a large time scale, and analyzing such data is not conducive to revealing the complicated rule changes that occur when correlating non-stationary and fluctuating time series data, such as coal prices and death tolls. In addition, the time lag of the mutual influence between prices and deaths has not been considered; thus, the influence of the time intervals and periodicity have not been estimated [7,8]. To date, warnings and preventive measures for coal mine accidents have not been applied in accordance with changes in coal prices. Hence, we aimed to study the features of the asynchronous correlation between coal prices and accident deaths on a smaller time scale. How does the asynchronous correlation between coal prices and coal mine deaths change, and which change patterns have a greater impact? How do different asynchronous correlation types influence each other? When one of the asynchronous correlation types changes, which type will play the intermediary role? What are the transmission rules, paths and distances among the variations of the asynchronous correlation as it fluctuates? To answer these questions and reveal the complex features of the asynchronous correlation, general statistical methods and time series models are not suitable. The theory of complex network aims to analyze the relationship between the elements in a network model derived from a real complex system. Visual graphic algorithms based on complex network theory were investigated to transform various types of time series into networks, in which the sequence data points and their connections over time are considered nodes and edges, respectively [9–14]. The important features and dynamic behavior of various real systems, including macroeconomics, geophysics, biology, fluid science etc., can be clearly understood by studying the topological structure and related parameters of the corresponding network models [15–25]. This theory provides new perspectives and methods for studying correlations in a number of real systems. For example, small-world characteristics, power-law distributions, clustering effects, etc. are found in economic systems [26–30]. Therefore, we can use the theory and method of complex networks to analyze the asynchronous correlation between coal prices and accidental deaths.

In this paper, we propose a method of studying the features of the asynchronous correlation between two time series of the China coal price index and the number of coal mine accident deaths. The China coal price index (**CCPI**) is a comprehensive index that provides an objective and timely description of coal economic operations and changes in coal market environment in China. The coal mine accidental death (**CMAD**) index directly reflects the status of coal mine deaths. The above questions will be answered by defining the small-scale and asynchronous correlations between two variables as different fluctuation modes and transforming these variables into different network models. To explore the features of the asynchronous correlation between the two variables, the complex network analysis method will be applied to identify the key statistical parameters, the network community characteristics and the evolution mechanisms.

## Materials and Methodology

### Materials

The **CMAD** data from 2012–2015 used in this paper were obtained from the governmental online accident inquiry system of the State Administration of Work Safety of China (http://media.chinasafety.gov.cn:8090/iSystem/shigumain.jsp), and the **CCPI** data from 2012–2015 were obtained from the official website of the Chinese National Coal Association (http://www.coalchina.org.cn/page/zt/120712/). The data were collected from publicly available databases, and all the data applied in this paper are freely available to other researchers.

**Fig 1:**
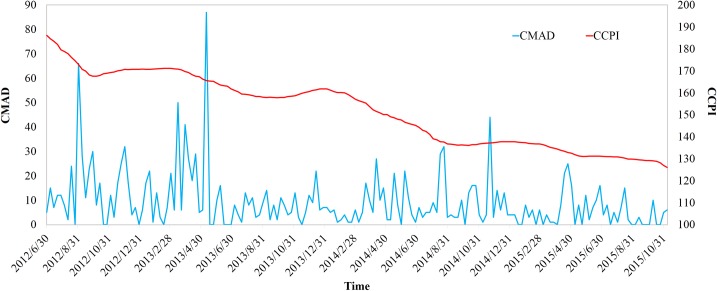
CMAD and CCPI series data from July 1, 2012 to November 8, 2015.

**CMAD** and **CCPI** data from July 1, 2012 to November 8, 2015 were selected and shown in Fig 1. Because the **CCPI** data are published weekly, the consistency of the time scale between the two time series had to be ensured. Thus, we aggregated the **CMAD** data into weekly data, and the two resulting series each contained 176 data points.

### Asynchronous scenarios

**Fig 2:**
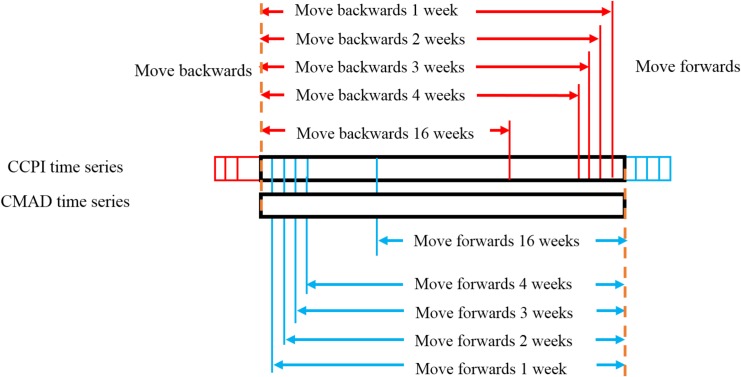
Construction process of the asynchronous scenarios.

Because the correlation between the **CCPI** and **CMAD** is not necessarily synchronous, different asynchronous scenarios were constructed provide additional information on the asynchronous correlation between the two studied variables. As shown in Fig 2, the **CMAD** was set as the benchmark, and then an adjustment process was implemented by the moving the **CCPI** forwards and backwards 16 weeks with a unit step of 1 week. Thus, a total of 33 asynchronous scenarios *L* with different time intervals and directions were obtained (the synchronous scenario is *L* = 0). If the **CCPI** moved forward, then the value of *L* was positive, whereas if it moved backwards, the value was negative.

### Fluctuating modes of the asynchronous correlation

To explore the features of the asynchronous correlation, the fluctuation modes were defined by the asynchronous correlation coefficient between the **CCPI** and **CMAD** within each asynchronous scenario, symbolization transition and coarse-graining process. The fluctuation modes denoted different patterns of asynchronous correlation change.

First, based on the Pearson correlation coefficient, the time delay factor was introduced to describe the correlation at different asynchronous scenarios. The asynchronous correlation coefficient $r_{xy}^{L}$ was calculated to quantify the degree of correlation between *x* (**CCPI**) and *y* (**CMAD**). The asynchronous correlation $r_{xy}^{L}$ at delay *L* was defined as follows:

$$r_{xy}^{L}=\frac{\sum_{i=1}^{n}\left(x_{i}^{L}-\bar{x^{L}})(y_{i}^{L}-\bar{y^{L}}\right)}{\sqrt{\sum_{i=1}^{n}{\left(x_{i}^{L}-\bar{x^{L}}\right)}^{2}}\sqrt{\sum_{i=1}^{n}{\left(y_{i}^{L}-\bar{y^{L}}\right)}^{2}}},\;L\in [-16,\;16]$$ (1)

where $x_{i}^{L}$ and $y_{i}^{L}$ are the time series values of the **CCPI** and **CMAD**, respectively, in the different asynchronous scenarios, $\bar{x^{L}}$ and $\bar{y^{L}}$ are the corresponding mean values, and *n* is the number of data points.

**Fig 3:**
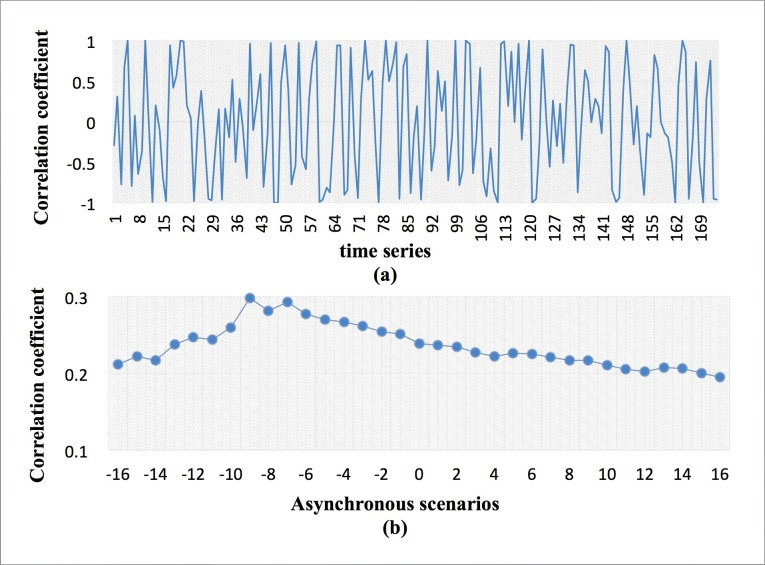
Correlation coefficient of the terms (*L* = 0) and overall correlation coefficient in the asynchronous scenarios.

Thirty-three sets of asynchronous correlation coefficients were calculated. Fig 3A shows that the asynchronous correlation (*L* = 0) between the two variables was not stationary and the fluctuation process was nonlinear, which is consistent with the features of other asynchronous scenarios. According to Fig 3B, the overall correlation varied among the different asynchronous scenarios.

Second, based on the value of $r_{xy}^{L}$, the asynchronous correlation was symbolized into different types according to its ‘degree’. After calculating the proportion of 20 isometric thresholds in the range of correlation coefficient, we discretized the correlation coefficients into 5 levels including strong positive and negative correlation, weak positive and negative correlation and no correlation. The symbols ${ACS}_{i}^{L}$ with different letters represent the corresponding degrees of asynchronous correlation.

$${ACS}_{i}^{L}=\left\{ \begin{array}{l} A,\;r_{xy}^{L}\in [\;0.8,\;1.0\;]\\ B,\;r_{xy}^{L}\in [\;0.3,\;0.8)\\ C,\;r_{xy}^{L}\in (-0.3,\;0.3)\\ D,\;r_{xy}^{L}\in (-0.8,\;-0.3]\\ E,\;r_{xy}^{L}\in \;[-1.0,\;-0.8], \end{array}\right.$$ (2)

**Fig 4:**
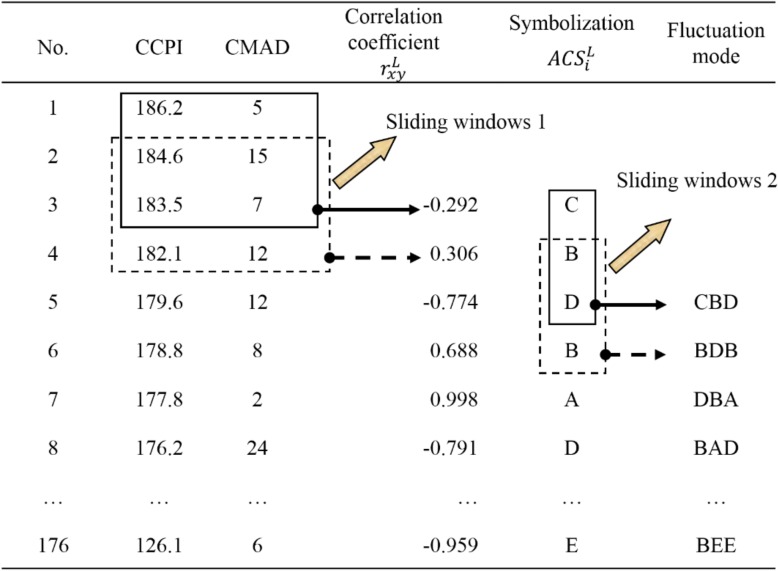
Coarse-graining process used to define the fluctuating modes (*L* = 0, *T* = 3, and *ω* = 3).

Third, in the coarse-graining process, we used the method of sliding windows to divide the asynchronous correlation coefficient sequences and symbol sequences into terms [31]. Note that the two time scales *T* and *ω* of sliding windows 1 and 2 should be determined reasonably and should not be too long or too short. After a comprehensive analysis, we selected the data points for *T* = 3, 4, and 5 weeks as the terms and created sliding data window 1 for the correlation coefficients with a sliding step of 1. Accordingly, the asynchronous correlation coefficient sequences contained 177 − |*L*| − *T* terms. Similarly, for *ω* = 3, the sliding data window 2 of the symbols could be built as terms with the same sliding step. Accordingly, the asynchronous correlation coefficient sequences contained 178 − |*L*| − *T* − *ω* terms. Fig 4 shows the process of defining the asynchronous correlation fluctuating modes (*L* = 0, *T* = 3, and *ω* = 3).

**Fig 5:**
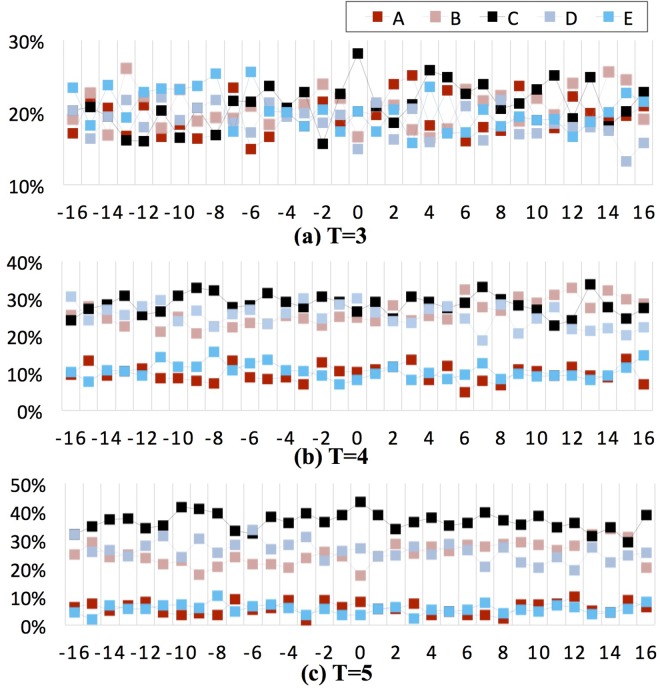
Proportion of the elements in the symbol sequence (*ω* = 3, *T* = 3, 4, and 5).

As shown in the three sub-plots (*T* = 3, 4, and 5) of Fig 5, the ordinate is the proportion of the elements in the symbol sequences and the abscissa is the asynchronous scenarios. The proportion of the elements that represent no correlation (black box) increased with increases in *T*, whereas the proportion of the elements representing strong correlations (bright blue and bright red box) decreased with increases in *T*. For *T* = 5, an obvious stratification in the different degrees of the correlation was observed. For *T* = 3, a significant difference was observed in the distribution of various correlation degrees between the different asynchronous scenarios.

### Asynchronous correlation fluctuating mode directed and weighted network

The preliminary comparison and selection in the coarse-graining process identified 33 sets of asynchronous correlation fluctuation mode sequences (*T* = 3, *ω* = 3) denoted as ${ACS}^{L}=\{{ACS}_{i}^{L}\},\;(L=-16,-15,\ldots ,16)$. For example, the fluctuating modes evolve into each other with time: ${ACS}_{i}^{0}=\{CDB\to BDB\to DBA\to ,\cdots ,BEE\}$. To clarify the rules underlying the development of the fluctuation modes, we constructed an asynchronous correlation fluctuating mode directed and weighted network (**ACFM-DWN**). To minimize the proportion without a correlation, 9 asynchronous correlation fluctuation mode sequences were selected.

**Fig 6:**
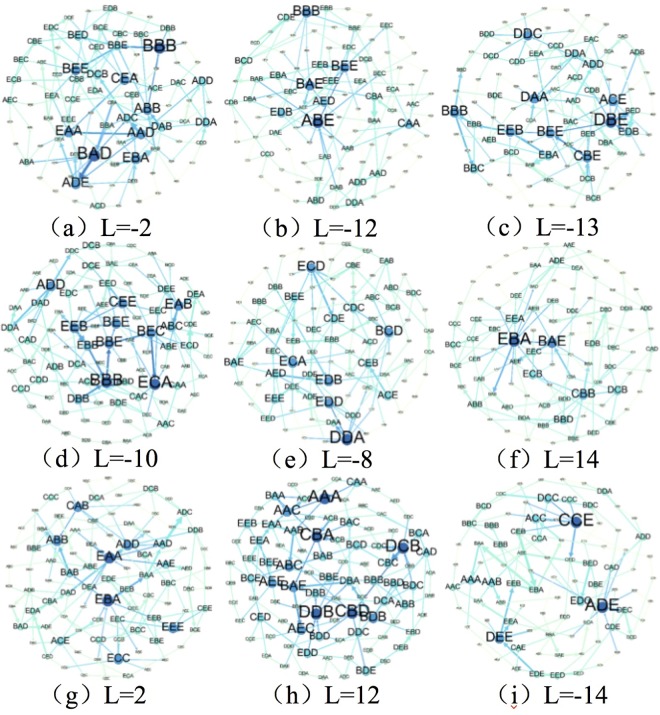
Asynchronous correlation fluctuating mode directed and weighted networks (ACFM-DWN) (*T* = 3, *ω* = 3). ***N*** and ***E*** are the number of nodes and edges respectively in each network. The greater the transitional impact, the larger the node in the network.

By transforming the fluctuation modes and their linkages into the nodes and edges of the networks, 9 complex network models of the **CCPI**-**CMAD** asynchronous correlation fluctuation modes were established (as shown in Fig 6).

## Results

As mentioned above, the 4 questions to be answered in this paper are managed by the complex network analysis method, which provides a number of useful parameters, including the node degree, the average path length, the clustering coefficient, and the structural hole hierarchy. Using these parameters, the statistics, change rules and evolution mechanisms of the asynchronous correlation between fluctuations in the **CCPI** and **CMAD** could be studied.

### Change rules and key fluctuation modes of the asynchronous correlation

How does the asynchronous correlation between coal prices and coal mine deaths change, and which change patterns have a greater impact? To answer these questions, a basic statistical description and the key fluctuation modes of the asynchronous correlation should be identified. For the 9 **ACFM-DWN** models in Fig 6, each node denotes one fluctuation mode representing the **CCPI**-**CMAD** correlation over a small time scale. The elements of the fluctuation mode symbol sequence were calculated statistically. Combined with the statistical results of the frequency distribution of the symbol elements in Fig 5A, an asynchronous correlation was observed between the **CCPI** and **CMAD**. Taking the analysis a step further, a comprehensive perspective of each of the asynchronous scenarios indicates that the correlation types include strong positive, strong negative, weak positive, weak negative, and even no correlation. Therefore, the correlation between the two variables was asynchronous and fluctuating. Certain features are briefly summarized as follows.

- In the asynchronous scenario *L* = 2, a strong positive correlation (with the highest frequency of 23.84%) occurred when the **CCPI** lagged the **CMAD** by 2 weeks. The proportion of weak positive correlations was 20.93%. Therefore, a more positive correlation occurred between the two variables in these asynchronous scenarios.
- In the asynchronous scenarios *L* = −14, −12, −10, *and* −8, the frequencies of strong negative correlation elements were the highest when the **CMAD** lagged the **CCPI** 14, 12, 10, and 8 weeks, and the proportions of the elements were 23.75%, 22.84%, 23.17%, and 25.30%, respectively. Therefore, a more negative correlation occurred between the two variables in these asynchronous scenarios.
- For the remaining 4 asynchronous scenarios, the frequency distribution of weak and no correlation elements accounted for more than 58.00%, whereas the maximum was 64.00%. Therefore, the degrees of correlation between the **CCPI** and **CMAD** in these 4 scenarios did not accurately explain the fluctuation process.

The key fluctuation mode of the asynchronous correlation indicated the mode that had a much higher impact in the **ACFM-DWN**, and fluctuation modes with a greater node strength were more likely to have a significant impact. The statistics of node strength, based on the transformation between modes, contains both the neighbors’ number of node and the weights between them [32]. Thus, the key fluctuation mode of the asynchronous correlation could be found by calculating and analyzing the node strength and the topological properties in the network model. The node strength *ns*_*i*_ is defined as the sum total of the weighted values of the edges connected to one node, which indicates the comprehensive local information of node. The node strength can be calculated using the following equation:

$${ns}_{i}=\sum_{j\in N_{i}}w_{ij},$$ (3)

where *N*_*i*_ is the total number of neighbors of node *i* and *w*_*ij*_ is the weight of edge from node *i* to node *j*, which is the frequency of transformation between them. In Fig 6, larger nodes indicate a greater node strength value.

Theoretically, there may be 125 types of fluctuation modes along with the possible combinations of letters. However, approximately 90 types of modes form nodes in each model. Table 1 shows the double logarithmic relationship between the weighted degree of the node and the transmission probability within the 5 **ACFM-DWN**s (*L* = 2, −8, −10, −12, and −14). The calculation indicated that the weighted degree node of the fluctuation modes in the networks obeys a power-law distribution [33]. In addition, the corresponding linear regression equations and coefficients of determination (*R*^2^) were obtained. According to the characteristics of the power-law distribution, we determined that these 5 networks were scale-free networks. Therefore, the frequencies of most of the fluctuation modes in the network models were low, and only a small fraction had a significant impact on the fluctuation of the asynchronous correlation. For example, the 7 modes ABE, BBB, BAE, BEE, CAA, AED, and EDB (shown in Fig 6B) play key roles in the fluctuation process and present a total transformation frequency of more than 16%. It means that the positive and negative correlation obviously exist between in asynchronous scenario *L* = −12. The direction and degree of correlation in key fluctuation modes generally represent the characteristic of asynchronous correlation under the corresponding time delay. Therefore, the trends of accidents will be obtained key fluctuation modes could be useful for determining the trends of accidents.

**Table 1:** Power-law distribution in the ACFM-DWNs.

Lag	Average path length	Fitted equation	*R*^2^
L = 2	6.732	*P(k)* = 2.7463 *k* ^-2.326^	0.848
L = -8	6.664	*P(k)* = 3.1551 *k* ^-2.441^	0.775
L = -10	7.027	*P(k)* = 1.7268 *k* ^-2.092^	0.632
L = -12	6.508	*P(k)* = 3.0628 *k* ^-2.594^	0.746
L = -14	7.402	*P(k)* = 4.3289 *k* ^-2.802^	0.801

### Community characteristics

How do different asynchronous correlation types influence each other? To answer this question, we must determine the fluctuation rules and the fluctuation modes that play central roles. In the fluctuation process of the **CCPI**-**CMAD** asynchronous correlation, certain fluctuation modes interact with each other, and changes in one fluctuation mode are always controlled by another mode. As shown in Fig 6, a certain amount of small network groups are observed in each **ACFM-DWN**. Accordingly, because the community structure is one of the most important topological properties in complex networks, it should be studied within the 5 **ACFM-DWN**s (*L* = 2, −8, −10, −12, and −14). Due to the network models with small scale, the method for mining the local close-knit structures with a relatively loose definition and higher resolution is needed [34–37]. In particular, a mixed method will be applied by combining the k-plex method with the clustering coefficient.

In any subgroup explored by the k-plex method, each node has *g* − *σ* linkages with the other nodes, where *g* is the number of nodes in a subgroup and *σ* is the adjustment parameter. With decreases of *σ* and increases of *g*, fewer subgroups could be explored, which indicates that there are more transformation forms of the nodes in the network [38]. The transmission probability between neighbor nodes in the subgroups can be analyzed by calculating the clustering coefficient of each node to identify the core node in the subgroup [39]. If the clustering coefficient of node is greater in subgroup, the transformation with other nodes is more closely and the core position is more significant.

$${CC}_{i}=\frac{E_{i}}{k_{i}(k_{i}-1)/2},$$ (4)

where *E*_*i*_ is the number of actual shared edges between neighbor nodes and *k*_*i*_(*k*_*i*_ − 1)/2 is the maximum number of shared edges.

Table 2 shows the subgroups of the 5 **ACFM-DWN**s, where *g* = 3 and *σ* = 2. Three to six subgroups were obtained within the 5 networks of the asynchronous scenarios. The clustering coefficient of each fluctuation mode is elaborated in Table 2, which confirms each subgroup’s correlation category and its core mode.

**Table 2:** Subgroups in the 5 ACFM-DWNs.

Lag	No.	Subgroup of fluctuation mode			Correlation category
**L = 2**	1	ABB(0.067)	BBE(0.083)	**BBB(0.500)**	**Weak positive** correlation
	2	ABB(0.067)	**BBA(0.167)**	BAB(0.050)	**Weak positive** correlation
	3	**CEE(0.083)**	**EEB(0.083)**	**EEE(0.083)**	**Weak negative** correlation
	4	ADB(0.167)	**DBA(0.500)**	BAD(0.083)	**Weak positive** correlation
	5	BCC(0.083)	**CCB(0.167)**	**CBC(0.167)**	**No** correlation
**L = -8**	1	EDD(0.067)	DDE(0.083)	**DDD(0.167)**	**Weak negative** correlation
	2	EDD(0.067)	DDA(0.024)	**DDD(0.167)**	**Weak negative** correlation
	3	ECD(0.033)	CDE(0.050)	**DEC(0.083)**	**Weak negative** correlation
**L = -10**	1	EBB(0.050)	**BBB(0.100)**	BBE(0.033)	**Weak positive** correlation
	2	**BBB(0.100)**	DBB(0.050)	BBD(0.083)	**Weak positive** correlation
	3	EEC(0.083)	CEE(0.033)	**ECE(0.500)**	**Strong negative** correlation
	4	DDA(0.083)	ADD(0.033)	**DAD(0.083)**	**Weak negative** correlation
	5	DEA(0.083)	**EAD(0.500)**	**ADE(0.500)**	**Weak negative** correlation
	6	EEB(0.033)	BEE(0.033)	**EEE(0.500)**	**Strong negative** correlation
**L = -12**	1	BBB(0.050)	**EBB(0.083)**	**BBE(0.083)**	**Weak positive** correlation
	2	ECD(0.167)	CDE(0.050)	**DEC(0.500)**	**Weak negative** correlation
	3	BEE(0.024)	**EEE(0.050)**	**EEB(0.050)**	**Strong negative** correlation
	4	**DDD(0.167)**	DDA(0.050)	ADD(0.050)	**Weak negative** correlation
**L = -14**	1	DEE(0.033)	**EEE(0.500)**	EEA(0.083)	**Strong negative c**orrelation
	2	**BAA(0.167)**	AAB(0.050)	AAA(0.050)	**Strong positive c**orrelation
	3	CCE(0.024)	DCC(0.050)	**CCC(0.500)**	**No** correlation
	4	CDE(0.083)	DEC(0.083)	**ECD(0.167)**	**Weak negative** correlation
	5	EBB(0.083)	BBC(0.083)	**BBB(0.500)**	**Weak positive** correlation

According to the results in Table 2, the correlation categories of the 5 subgroups (*L* = −14) were diverse. Thus, in this asynchronous scenario, specific rules cannot be applied to the fluctuation of the **CCPI**-**CMAD** correlation.

In addition, in the context of *L* = 2, three out of the five subgroups mainly showed weak positive correlation in fluctuation, and the **CCPI** will change with a same trend as the **CMAD**. Combined with the actual data, the **CMAD** stably fluctuated with fewer than 25 persons and the **CCPI** slowly increased by approximately 1% after 2 weeks, whereas **CMAD** increased sharply with more than 25 persons and the **CCPI** decreased continuously and rapidly by approximately 2.5% after 2 weeks.

Finally, for *L* = −8, three subgroups were all characterized by weak negative correlations; for *L* = −10, four out of six subgroups showed obvious negative correlations; and for *L* = −12, three out of five subgroups primarily showed negative correlations. Therefore, the **CMAD** will follow an opposite trend from that of the **CCPI** when the **CCPI** shows an 8, 10, and 12 week lag. A comparison of the actual data showed that for *L* = −8, when the **CCPI** declined by approximately 0.7%, the **CMAD** increased by approximately 7 persons after 8 weeks; whereas when the **CCPI** increased at a rate between 0.2% to 1.8%, a significant reduction of the **CMAD** occurred after 8 weeks, and the **CMAD** was maintained at less than 11 persons. For *L* = −10, when the **CCPI** decreased at a rate between 0.7% and 3.9%, a surge in the **CMAD** of 14 to 24 persons was observed after 10 weeks; whereas when the **CCPI** increased at a rate of approximately 1.8%, the **CMAD** showed a distinct reduction and remained at less than 5 persons after 8 weeks. For *L* = −12, when the **CCPI** decreased by 0.4% to 2.2%, the **CMAD** increases greatly by approximately 6–20 persons after 12 weeks; whereas when the **CCPI** increased by 0.2%-0.8%, the **CMAD** decreased and remain at approximately 4 persons after 12 weeks.

By exploring and mining the subgroups and their core modes in the networks, differences in the correlation categories were observed among subgroups; however, frequent transformations occurred between the fluctuation modes within the same subgroup. Consequently, different fluctuation modes did not show a disorderly transform into each other and their transformations are centered on several modes, with each type of subgroup fluctuating around one core mode.

Furthermore, we found that a weak positive correlation occurred between the **CCPI** and **CMAD** with positive values of *L*, which was primarily because after the large and sharp increases in the number of deaths caused by an accident, large-scale reform measures were performed. Subsequently, the production rates in a large number of coal mining enterprises decreased, thereby leading to an overall decline in the coal economy. However, a strong and weak negative correlation was observed between the **CCPI** and **CMAD** in the asynchronous scenarios with negative values of *L*, which was caused by the decline of the **CCPI**. Safety investments are limited by their reduction of economic benefits to the enterprises. Therefore, the asynchronous negative correlation rule has a reference value in coal mining accident prevention, and based on the changing trends of the core fluctuation modes and the length of the lag, an adequate amount of time should be available to implement prevention and control measures for accidents.

### Transmission medium

When one of the asynchronous correlation type changes, which type plays the intermediary role? To answer this question, we analyzed the characteristics of the structural hole of the networks to determine the fluctuation modes that act as the transmission medium in the **ACFM-DWN**s. The presence of structural holes provides the middle-position fluctuation modes with key communication functions and allows them to control the transmission process of the correlation fluctuation to a great extent. Generally, the Coleman-Theil disorder index is used to measure the hierarchy of structural holes for each node in network, and the index is defined as *H*_*i*_.

$$H_{i}=\frac{\sum_{j}\left(\frac{C_{ij}}{C/N}\right)\ln\left(\frac{C_{ij}}{C/N}\right)}{N\;\ln\left(N\right)},$$ (5)

where *N* is the set of the individual networks consisting of node *i* and its neighbors and *C*/*N* is the mean value of all node constraints *C*_*ij*_. The node constraint *C*_*ij*_ is the ability level of node *i* to use the structural hole in its individual network, and it is expressed as follows [40]:

$$C_{ij}={\left(p_{ij}+\sum_{q}p_{iq}p_{pj}\right)}^{2},$$ (6)

where *p*_*iq*_ is the proportion of the relationship between nodes *i* and *q* in the total relationships.

**Fig 7:**
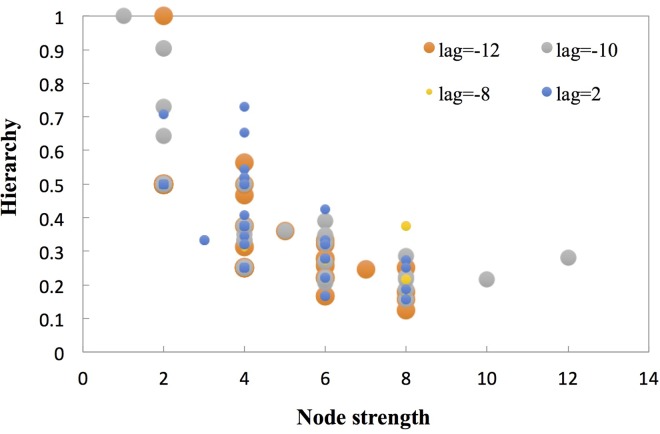
Distribution between the node strength and the hierarchy (*L* = −12, −10, −8, 2).

Fig 7 shows that a small fraction of the fluctuation modes possess a higher structural hole hierarchy; however, low node strengths occur in each **ACFM-DWN** (*L* = −12, −10, −8, 2). For DBA, BBB, ADB, and BBA in **ACFM-DWN** (*L* = 2), the highest hierarchy of the structural hole reaches 0.731. Accordingly, although certain of these nodes are not the major modes, they all likely play important roles in the transformation of the weak positive correlation between the **CCPI** and **CMAD**. Moreover, the appearance of these transmission media indicate whether or not this period is conducting a significant transformation; subsequently, the next fluctuation mode can be predicted via the transmission probability.

### Transmission periodicity and path

What are the transmission rules, paths and distances among the different variations within the fluctuation of the asynchronous correlation? To answer this question, the periodicity of the asynchronous correlation coefficient and the shortest distance between each pair of fluctuation modes were determined. By performing a spectral analysis and calculating the shortest path length, the periodic features and major fluctuating patterns of the asynchronous correlation were studied.

**Fig 8:**
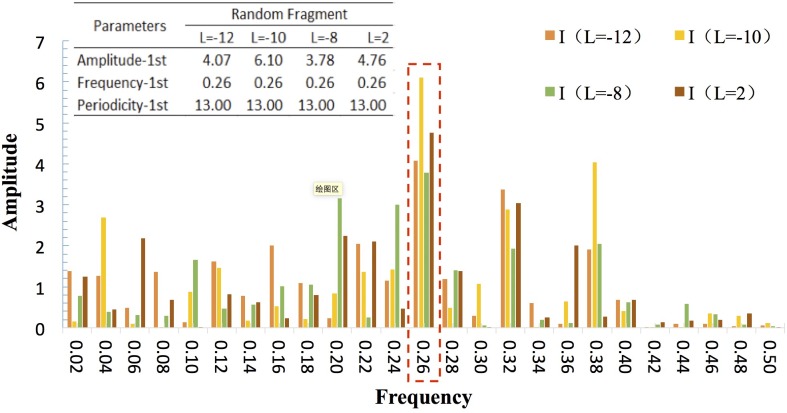
Spectral map (*L* = −12, −10, −8, 2).

The periodic features of the **CCPI**-**CMAD** asynchronous correlation could be illustrated by a spectral analysis, whose core function is to express the time series as a superposition of sines and cosines with different frequencies. Fig 8 shows the spectral analysis results of the 4 sets of correlation coefficient time series (*L* = −12, −10, −8, 2) randomly selected by a length of 50 weeks. The results indicate that periodicity in the asynchronous scenarios generally appeared in the superposition after 13 weeks. For instance, in the asynchronous scenario (*L* = −10), the periodicity of strong negative correlation is 13 weeks, which means the situation that CMAD would increase after CCPI decrease 10 weeks happens once every 13 weeks. From the perspective of accident prevention, the 13 weeks can be regarded as the periodicity of implementation of normalized control measures.

On the basis of the periodicity obtained by spectral analysis, the average path length indicates the transformation distance and path between nodes [41], and the shortest path length is the minimum number of edges between any pair of nodes. We can therefore improve the accuracy of the periodicity length and the specific form of the modes on the path. The average path length of the network *APL* was the average value of the shortest path lengths of all pairs of nodes, and it was defined as follows:

$$APL=\frac{1}{n(n-1)/2}\sum_{i\geq j}d_{ij},$$ (7)

where *d*_*ij*_ is the minimum number of edges from node *i* to node *j*.

**Fig 9:**
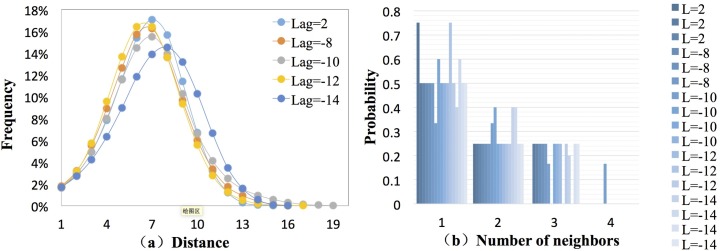
Frequency of the distance and transmission probability of the major fluctuating modes.

In Fig 9(A), a similar distribution pattern of the average shortest path length appears in 5 networks, and the shortest path and longest path are 1 and 19, respectively. The distances with a high frequency that were observed in than 40% of cases in the **ACFM-DWN**s are 6, 7, and 8. The average path length *APL* of the 5 networks was approximately 7 (Table 1). Thus, if a fluctuation mode transformed to another mode, 6 or 8 types were involved in the process. Because of the low proportion of paths with under 2 or above 11 steps, different modes can rarely complete the transformation using these distances. Fig 7(B) presents the transmission probabilities of the major fluctuating modes within the 5 networks. Although the number of neighboring nodes of these major nodes was only 2 to 4, there were only 1 or 2 neighbors with higher transmission probabilities. Consequently, the transmission probabilities could be used to conclude the fluctuation form in next period and applied to build the risk estimation model combined with the severity of specific modes. The corresponding control policy would be refined according to the risk level.

## Discussion and Conclusions

In this paper, our major goal was to study the features of the asynchronous correlation between the **CCPI** and **CMAD** over a small time scale using the method of complex network. The 33 asynchronous scenarios of the double-variable time series were first constructed. The fluctuation mode of the asynchronous correlation was defined by the asynchronous correlation coefficients, symbolization, and coarse-graining process. Based on a mode-representing fragment, the correlation between the **CCPI** and **CMAD** was asynchronous and characterized by fluctuation. After introducing the complex network theory, the properties of the correlation fluctuating mode directed and weighted networks were studied. The approach in this paper is suitable for analyzing the asynchronous correlation between the coal mine price index and accident deaths, and it can also be used for other coal mine economic indices, which could provide value for coal mine accident pre-warning systems. The features of the asynchronous correlation between the coal price index and accidental deaths could provide an effective reference for governmental agencies tasked with executing safety supervision measurements. The accident prevention strategies could be divided into various degrees that correspond to the fluctuation modes of varying degrees of importance to the asynchronous correlation. When key fluctuation modes are observed, a strong prevention strategy is required because the asynchronous correlation will exhibit a more complex pattern of changes, and certain changes may cause an increase in the number of accidental deaths. By exploring the sub-groups in which a small fraction of fluctuation modes influence each other at a high frequency, governmental agencies could forecast the changing trends of accidental deaths based on the changing forms of the **CCPI**. In particular, a continuous decline in the degrees of variation in the **CCPI** may cause a sudden rise in the **CMAD** within different ranges. However, because there is at least an 8 week lag before an accident occurs, the governmental agency will have sufficient time to take preventive measures. The fluctuation modes with high structural hole hierarchies indicate the role of the transmission medium, which can warn governmental agencies that changes will occur in the fluctuation form of the **CCPI**-**CMAD** asynchronous correlation. Calculating the periodicity and the shortest path and length indicates the impact pattern of the **CCPI** and the corresponding changing rule in the **CMAD** within a short recurrent period and can provide a reference for accident pre-warning policies.

Certain issues must be investigated in greater detail in future research. Firstly, the method in this study fits for analyzing the asynchronous correlation features between two variables. However, there must be other indices who are more suitable for pre-warning **CMAD**. So, a more appropriate approach and determining coefficient for screening better pre-warning index are well worth in further study. We will take more network measures, such as clustering coefficient entropy and network cross-transitivity into consideration [42,43]. Secondly, according to the method and results of this study, a pre-warning model for coal mine accidental deaths can be established based on asynchronous correlation features of multivariate time series. More features would be adopt in studying the estimation method, acceptable criteria, and control policy of **CMAD** in a view of risk pre-warning. It is different from the general risk estimation models because the factors containing actual value of sub-variables, time delay, influence degree, transmit distance and probability, periodicity, time-scale of pre-warning and so on are considered. The influence of information losses could be avoided in the fluctuation process of the multivariate asynchronous correlation, and the pre-warning information of coal mining accidental deaths could be more accurately and detailed. Then, the authorities responsible for overseeing the work safety environment in the coal mining industry could take preventive measures that are consistent with specific changes in the **CCPI** to control for large-scale coal mine accidents before they occur. In addition, this paper may also inspire readers by providing a new approach for the study of asynchronous correlations between two time series.
